# Epigenetic modifications as therapeutic targets in atherosclerosis: a focus on DNA methylation and non-coding RNAs

**DOI:** 10.3389/fcvm.2023.1183181

**Published:** 2023-05-25

**Authors:** Hashum Sum, Alison C. Brewer

**Affiliations:** School of Cardiovascular and Metabolic Medicine & Sciences, King’s College London British Heart Foundation Centre of Excellence, London, United Kingdom

**Keywords:** atherosclerosis, DNA methylation, microRNAs, epigenetics, stem cell therapy, CRISPR/Cas9, TET2, DNMT3a

## Abstract

Significant progress in the diagnosis and treatment of cardiovascular disease (CVD) has been made in the past decade, yet it remains a leading cause of morbidity and mortality globally, claiming an estimated 17.9 million deaths per year. Although encompassing any condition that affects the circulatory system, including thrombotic blockage, stenosis, aneurysms, blood clots and arteriosclerosis (general hardening of the arteries), the most prevalent underlying hallmark of CVD is atherosclerosis; the plaque-associated arterial thickening. Further, distinct CVD conditions have overlapping dysregulated molecular and cellular characteristics which underlie their development and progression, suggesting some common aetiology. The identification of heritable genetic mutations associated with the development of atherosclerotic vascular disease (AVD), in particular resulting from Genome Wide Association Studies (GWAS) studies has significantly improved the ability to identify individuals at risk. However, it is increasingly recognised that environmentally-acquired, epigenetic changes are key factors associated with atherosclerosis development. Increasing evidence suggests that these epigenetic changes, most notably DNA methylation and the misexpression of non-coding, microRNAs (miRNAs) are potentially both predictive and causal in AVD development. This, together with their reversible nature, makes them both useful biomarkers for disease and attractive therapeutic targets potentially to reverse AVD progression. We consider here the association of aberrant DNA methylation and dysregulated miRNA expression with the aetiology and progression of atherosclerosis, and the potential development of novel cell-based strategies to target these epigenetic changes therapeutically.

## Background

Epigenetics is the study of mechanisms that regulate mitotically stable patterns of gene expression and hence cellular phenotype without altering the genomic sequence of the cell. The field has converged upon the locus-specific regulation of chromatin structure and accessibility by DNA methylation, the covalent and non-covalent modification of histones together with non-coding RNA-based mechanisms (1). Epigenetic changes are typically reversible, enzymatically-regulated processes which are modulated by the intracellular milieu. Consequently, they are critical drivers of cellular differentiation, development and homeostasis (2) but can lead to disease states when influenced by pathological environmental factors (3). Understanding the mechanistic causes and pathological consequences of aberrant epigenetic regulation might therefore offer the possibility of novel curative therapeutic treatments for human diseases. This is particularly true in the case of atherosclerosis, where it remains the case that current (predominantly lipid-lowering) treatments can only lessen, but not reverse, disease progression (4).

### DNA methylation and atherosclerosis

DNA methylation in mammals usually occurs at the 5th position of cytosine (5mC) in the context of CpG dinucleotides and is controlled by enzymatic systems which add or remove methyl groups, termed writers and erasers. CpG dinucleotides are distributed unevenly across the genome, and regions rich in CpG dinucleotides are known as CpG islands. Characteristically, CpG islands have a GC content >50% and are at least 200 bp in size. They contain around 7% of total CpG dinucleotides present in the human genome and are commonly found within promoter regions such that 60% of human gene promoter sites contain CpG islands (5). The DNA-methyl transferase (DNMT) family of enzymes add methyl groups to (predominantly cytosine) residues, while methyl groups can be removed passively via DNA replication or actively via the sequential oxidation of the methyl group, catalysed by the family of three Ten Eleven Translocase enzymes (TET1/2/3), producing 5-hydroxymethyl-cytosine (5hmC), 5-formyl-cytosine (5fC) and 5-carboxyl-cytosine (5caC). 5hmC is the most stable (and therefore most abundant) of these oxidation derivatives, while both 5fC and 5caC can be actively removed by base excision repair (BER) mechanisms to restore unmodified cytosines (6). The covalent modification of DNA affects both the structure of chromatin and the affinity for DNA-binding *trans*-acting factors and hence is a critical regulator of transcriptional gene expression (7). DNA methylation is usually associated with transcriptional repression and serves as a major cellular regulatory mechanism. It has numerous well-characterised roles across the biological spectrum, from embryonic development to cellular ageing and death and the manifestation of numerous diseases (8). By contrast, the precise functions of the individual TET-generated oxidised derivatives remain to be fully elucidated. Nevertheless, with the generation of TET mutants that uncouple these sequential oxidative steps, distinct regulatory functions for 5hmC, 5fC and 5caC are now becoming uncovered (9, 10).

Studies have shown that DNA methylation signatures are highly dynamic and can be influenced by different types of environmental stimuli that the individual had been exposed to, such as those known to be risk factors for the development of AVD (11, 12). In this regard it is noteworthy that the activities of both the DNMTs and TETs are known to be subject to modulation by risk factors associated with atherosclerosis, including oxidative stress and hyperglycaemia associated with diabetes (13, 14). Functional phenotypic changes may occur because of methylation-driven alterations in gene expression leading to improper regulation of homeostatic biochemical pathways. Many studies have investigated both changes in global levels of methylation, and changes in methylation at specific CpGs which link to vascular diseases including atherosclerosis. At the global level, genome-wide hypomethylation appears to associate with atherosclerosis, while hypermethylation of some specific genes are observed as the disease progresses (15)*.* Chronic inflammation and the development of fatty plaques are hallmarks of atherosclerosis. Perhaps accordingly, aberrant DNA (hyper)methylation has commonly been observed, associated with downregulation of key proteins that are essential in regulating the inflammatory response or those that are involved in the modulation of lipid pathways (Table 1). It should be noted that most of these studies to date have been retrospective and the methylation changes observed may therefore be a consequence rather than a cause of the pathologies. Crucially however, recent epigenome-wide association studies (EWASs) designed to investigate the association between incident CVD events associated with stable or unstable atherosclerotic plaques (such as stable coronary heart disease and myocardial infarction) have begun to identify characteristic methylation signatures which may be predictive of future risk and therefore causal with respect to cardiovascular outcomes (44). Reversing specific aberrant methylation marks, potentially in combination with other treatments, therefore represents a promising future clinical therapeutic strategy.

**Table 1:** Vascular disease-related genes that are regulated by DNA methylation.

Gene	Functional role of gene	Cells/tissue	Methylation status	Disease	Experimental condition	Effect of mutation	Reference
ABCA1	Modulator of HDL-C concentrations	Peripheral blood	Hyper	Atherosclerosis-induced coronary artery disease	Familial hypercholesterolemia	Increased risk from coronary artery disease	(16–18)
KLF2	Endothelium-dependent vascular homeostasis	Endothelial cells	Hyper	Atherosclerosis-induced coronary heart disease	Hypercholesterolemia	Impaired endothelial function Pro-inflammatory	(19, 20)
p66shc	Cellular redox	Endothelial cells	Hypo	Atherosclerosis/cholesterol-induced endothelial cell dysfunction	Hypercholesterolemia	Endothelial dysfunction Upregulation of ICAM1 Subsequent infiltration of monocytes Pro-coagulant Excess ROS production and pro-inflammatory	(21–23)
eNOS	Leads to the production of nitric oxide	Vascular smooth muscle cells	Hyper	Atherosclerosis/pulmonary hypertension	Pharmacological inhibition of HDAC using TSA	Decreased expression of eNOS is speculated to lead to endothelial dysfunction	(19, 24)
BAX, AMXA5, cIAP-1	Pro-apoptotic (BAX, AMXA5) Anti-apoptotic (cIAP-1)	Endothelial cells	Hypo = BAX, AMXA5 Hyper = cIAP-1	Atherosclerosis	Ox-LDL model	Increased apoptosis in advanced atherosclerosis	(25)
PDGF	Cell proliferation and migration	Aorta smooth muscle cells/human umbilical vein ECs (HUVECs)	Hypo	Atherosclerosis	Hyperhomocysteinemia	HHcy-induced vascular dysfunction via PDGF upregulation SMC proliferation	(26–28)
ER-α	Athero-protective e.g., combines with estrogen to generate NO in ECs; supress VSMC proliferation	Vascular smooth muscle cells	Hyper	Atherosclerosis	Hyperinsulinemia Coronary atherectomy samples	VSMC pro-proliferation	(29–32)
ER-β	Anti-cell proliferative	Aortic endothelial and smooth muscle cells	Hyper	Atherosclerosis	Atherosclerotic patients	Contributes to the development of atherosclerosis and vascular ageing	(33)
IGF2	Regulates placental development and fetal growth	Vascular smooth muscle cells	Hyper	Atherosclerosis	Homocysteine (Hcy)-induced	Increased vascular smooth muscle cell proliferation	(34)
FOXP3	Maintenance of the suppressive properties of Tregs	Peripheral blood Treg cells	Hyper	Atherosclerosis	Using shRNA to silence DNMT3b in ApoE mice	Led to DNMT3b accelerated atherosclerosis Down-regulation of Tregs	(35–37)
KLF4	Anti-inflammatory atheroprotective transcription factor	Endothelial cells	Hyper	Atherosclerosis	Disturbed flow	Promotes proliferation Promotes macrophage M1 activation by suppressing KLF4 expression	(38–40)
CETP	Facilitates the exchange of triglyceride to cholesterol esters	Peripheral blood	Hyper	Atherosclerosis	Familial hypercholesterolaemia	Hypermethylation of CETP is associated with lower total cholesterol and LDL-C levels	(41)
SMAD7	Tumorigenesis, inflammation, and fibrosis	Peripheral blood^13^/primary cultured human umbilical vein smooth muscle cells (HUVSMCs)^14^	Hyper	Atherosclerosis	Atherosclerotic patients	Increase in SMAD7 methylation led to the increase in Hcy levels, a known atherosclerotic marker	(42, 43)

In considering the aetiology, development and potential treatment of AVD, it is crucial to identify and fully understand the specific roles of different cell types. The development of atherosclerosis involves the dysregulation of vascular cells (endothelial, vascular smooth muscle and adventitial fibroblasts) (45), together with haematopoietic cells of both the innate and adaptive immune system (46). Most associations between aberrant methylation and (either retrospective or incident) cardiovascular events have been observed in total circulating blood cells as this can be obtained in the most non-invasive way. However, it is possible to isolate and purify both sub populations of leukocytes and the small numbers of circulating endothelial cells (ECs) (47) and minimally invasive cell biopsy techniques are also being developed to isolate small numbers of cardiovascular cells (48, 49). This, together with the use and development of microfluidic techniques to enable “omic” analyses, including DNA methylation maps of single-cells selected from a tissue (50), will enable a better understanding of the tissue-specific roles of methylation changes in the development of AVD.

### DNA methylation and clonal expansion of somatic cell mutations in AVD.

Age is a potent and independent risk factor for atherosclerosis (51), in part due to the age-related accumulation of somatic mutations. Crucially, the clonal expansion of haematopoietic stem cells harbouring mutations (which confer a selective proliferative and/or survival to the cell) can result not only in haematopoietic malignancy but also an increased risk for CVD including atherosclerosis (52). Thus, atherosclerotic lesions typically display clonal growth and are postulated to be able to arise from neoplastic processes consequent upon critical somatic mutations within expanded haematopoietic cell populations (53). Strikingly, approximately 50% of all mutations which result in the clonal expansion of haematopoietic cells are within genetic loci of specific members of the DNA methylation-regulatory genes; *Dnmt3a* and *Tet2* (54). Further, whole-exome sequencing of samples obtained from coronary heart disease patients revealed that clonal hematopoietic mutations in these genes conferred a significantly greater (approximately 2-fold) risk of atherosclerosis-associated incident heart disease compared to patients without such mutations (55). These data suggest that loss of function of these genes provides (potentially tumour-promoting) selective advantage and clearly underline the functional significance of DNA methylation in the development of AVD. The causal effect of both DNMT3A and TET2 loss-of-function within myeloid cells upon clonal expansion and the development of atherosclerosis has also been demonstrated unequivocally in atherosclerosis-prone mice (56–58). Thus, in bone marrow transplantation studies, DNMT3A- and TET2-deficient haematopoietic stem/progenitor cells (HSPCs) were both shown to expand preferentially (and most notably into the macrophage population) leading to a marked increase in atherosclerotic plaque size (56, 58). Strikingly, despite exerting opposing catalytic effects with respect to DNA methylation, the lack of either DNMT3A or TET2 in these mice resulted in similar downstream transcriptomic and pathogenic cellular effects. This perhaps highlights the crucial importance of correctly balanced methylation and demethylation and should be taken into consideration in designing therapeutic strategies.

It is now well-recognised from studies in mice that vascular smooth muscle cells (VSMCs) within the atherosclerotic plaque arise from the clonal expansion of a few select cells within the medial arterial wall and although not conclusively proven, much evidence suggests that this is also the case in the human pathology (53). This raises the question as to whether similar mechanisms might underlie the clonal expansion of VSMCs in atherosclerosis to those which drive the expansion of (age-related) somatic cell mutations in haematopoietic cells. VSMCs in atherosclerosis exhibit considerable plasticity and can adopt phenotypes resembling foam cells, macrophages, mesenchymal stem cells and osteochondrogenic cells (59). Evidence suggests that DNA methylation is a mediator of phenotypic switching in VSMCs (60) and that the catalytic (5-hmC-generating) activity of TET2, in particular, plays an active role in this (61). Whether clonally-expanded VSMCs in atherosclerotic plaques commonly harbour somatic mutations in *Tet2* or *Dnmt3a* is not currently known, but is a clear possibility.

The involvement of aberrant DNA methylation in ECs, associated with atherosclerotic development has also been demonstrated. DNMT-dependent changes in EC DNA methylation, associated with disturbed flow and endothelial inflammation, have been shown *in vitro* and in animal models *in vivo* (38, 62) and a role for EC-expressed TET2 in atherosclerosis progression has been demonstrated in mice (57). Although technically challenging to demonstrate, recent studies involving single-cell sequencing have also identified clonal expansion of ECs, associated with cardiovascular pathologies (63–66). Further, segmental endothelial dysfunction (ED) (particularly associated with disturbed flow at the branch point of arteries) is a significant contributor to arterial remodelling resulting in atherosclerosis (67) although it remains to be determined whether this is due to a clonally-expanded EC population. The possibility that circulating endothelial progenitor cells (EPCs, derived from the haematopoietic stem cell population) might be recruited, incorporated and expanded into atherosclerotic lesions has been widely investigated (68). The origin and functional significance of these cells remains highly controversial (69). Nevertheless, in a comparative mutational profiling study, common somatic mutations in *tet2* were identified in some subjects between a mature population of these circulating endothelial (CD105/146^+ve^/CD45^−ve^) cells and CD34^+ve^ HSPCs, strongly suggesting the existence of a common precursor cell (70). This raises the possibility that critical somatic mutations, observed within expanded haematopoietic cell populations, may also impact upon EC function in the development of the atherosclerotic plaque. Again, it would clearly be of interest to determine whether mutations in DNA methylation modifying enzymes (TETs or DNMTs) associate with any clonal expansion of ECs in the context of atherosclerosis.

### Cell-based approaches to target aberrant DNA methylation

Given the clear association of DNA methylation changes and AVD, therapeutic strategies involving natural and synthetic pharmacological inhibitors of epigenetic modifications have, and continue to be, intensely investigated (71, 72). Further, the use of methyl-containing nutrients, notably folic acid and methionine, particularly in the prevention of CVD (including atherosclerosis), is of clear clinical benefit (73). However, more targeted strategies are required to reverse specific methylation changes shown to be causal in the disease.

Genetically-programmed cell-based strategies to both generate disease models and treat atherosclerosis are increasingly being investigated and developed. Gene editing, in particular using the clustered regularly interspaced palindromic repeat (CRISPR)-associated protein (CRISPR/Cas9) system is now widely implemented towards the goal of correcting specific mutations to treat a wide range of diseases (74). Many experimental studies have demonstrated the proof of principle that the correction of a single gene defect can alleviate the severity of atherosclerosis in animal models [reviewed in (75)]. With regard to DNA methylation, mutations in *Tet2* and *Dnmt3a*, associated with haematopoietic cell clonal expansion and CVD might therefore be considered therapeutic targets for correctional gene editing. An increasingly popular strategy for delivering edited genes for targeting cardiovascular dysfunction *in vivo*, in is by using some form of stem cell, such as mesenchymal stem cells (for example, haematopoietic stem cells), embryonic stem cells or induced pluripotent stem cells (iPSCs) as a vector, all of which have associated advantages and drawbacks [reviewed in (76)]. iPSCs derived from the patient offer advantages in terms of availability and reduced immuno-reactivity but have previously proved difficult to engineer for CRISPR/Cas9 action. However, a recent study has shown that this could be overcome following lentiviral transduction in an iPSC-derived macrophage model with almost 100% efficiency (77). There is also an increasing use of the large variety of mesenchymal stem cells that can be derived from the umbilical cord (UC-MSCs). These also have advantages of low immunogenicity, easy collection and isolation together with high paracrine potential (78).

A clear problem with any cell-based gene delivery system for addressing cardiovascular abnormalities, is that the site of dysfunction often involves multiple cell types and restoration of normal function may require site-specific changes in tissue architecture. In the case of the corrections of *Tet2* and *Dnmt3a* mutations within haematopoietic cells this may not present as an issue. However, as suggested above, mutations within these genes will also possibly be found to associate with EC and/or VSMC pathology. In this context, it is promising that ECs derived from EPCs *in vitro* can occupy the perivascular space when introduced into immuno-suppressed mice and anastomose with the host vasculature (79). Further, bone marrow-derived stem cells can also traverse the vessel walls of atherosclerotic plaques and form the smooth muscle cells that are enriched there in this condition (80).

In addition to genetic editing, the CRISPR/Cas9 system has been developed to edit epigenetic (DNA and histone) marks. By fusing “dead” Cas9 to the catalytic domains of epigenetic writers and erasers, epigenetic changes can be introduced at specific sites, guided by CRISPR guide-RNAs (gRNAs) (81). Both site-specific DNA methylation and demethylation, by fusing to DNMT and TET catalytic activity respectively, has been achieved in mammalian cells (82, 83). Moreover, the CRISPR/Cas9 technology has been adapted to target multiple sites simultaneously, with the use of a polycistronic-tRNA-gRNA (84). Going forward, as (tissue-specific) methylation signatures that are shown to be predictive and causal in atherosclerosis become identified, these would represent potential targets of epigenetic editing in appropriate stem cell-based therapies.

### MicroRNAs and AVD

Approximately 99% of the human genome does not encode proteins (85). Although once considered to be “junk DNA”, much of it is now understood to be highly transcribed into different sub-categories of long and short non-coding RNAs (ncRNAs) with regulatory and structural functions (86). MicroRNAs (miRNAs) are a class of short (18–26 nucleotides) ncRNAs which act as post-transcriptional regulators of gene expression (87). More than 1,500 miRNAs, comprising about 1%–5% of the human genome have so far been annotated (88, 89) which are estimated to regulate more than 60% of the mRNA transcriptome (90). They act by binding to a homologous sequence (typically within the 3′ untranslated region) of their target gene(s) and destabilising the mRNA and/or repressing translation [see (91, 92) for detailed reviews of their synthesis and mechanisms of silencing]. They therefore affect protein levels and hence cellular phenotype without modifying the primary DNA sequence and are thus considered as epigenetic modulators. A single miRNA may suppress the expression of multiple functionally-related genes in critical cellular signalling pathways (93). Consequently, miRNAs have emerged as important regulators of physiological processes in development and cellular homeostasis (94). Further, their dysregulation is increasingly shown to be associated with pathological states, most notably cancers, in many cases causally (95). Accordingly, many miRNAs are now known to play critical roles in normal cardiovascular physiology and to be key players in the development of CVD, including atherosclerosis (96, 97). A table of key miRNAs associated with functions relevant to atherosclerosis development and/or known to be misexpressed in atherosclerosis is shown (Table 2).

**Table 2:** Selected miRNAs dysregulated in atherosclerosis and/or involved in pathways relevant to atherosclerosis development.

miRNA	Cell/Tissue type	Target mRNA	Function and/or association with disease	Reference
miR-1	Blood		Expression associates with subclinical atherosclerosis. Induces VSMC differentiation	(98)
	VSMCS	Klf4 and Pim1		(99, 100)
miR-10a	VSMCs	HDAC4	Negative regulator of SMC differentiation	(101)
	Serum	GATA6	Represses inflammatory signalling	(102)
miR-10b	ECs	LTBP1	Deficiency caused resistance to atherosclerosis	(103)
miR-19a	ECs	Cyclin D1	Mediates the suppressive effect of laminar flow on cell cycle progression	(104)
	VSMCs	RHOB	Promotes VSMC proliferation	(105)
	B Cells	IL-10	Suppression of IL-10-mediated immunomodulation	(106)
miR-19b	ECs	SOCS3	Activates perivascular adipose tissue	(107)
	ECs	PGC-1α	Overexpression results in endothelial injury in atherosclerosis	(108)
	Macrophages	ABCA1	Promotes cholesterol accumulation and atherosclerosis	(109)
	Macrophage-derived exosomes	JAZF1	Promotes atherosclerosis	(110)
miR-21	Monocyte/macrophages	TNFa	Promotes anti-inflammatory signalling	(111)
	VSMCs		Regulation of cell proliferation.	(112)
miR-22	Macrophages	IRF5	Inhibition promoted atherosclerotic plaques	(113)
miR-26a	VSMCs	SMAD-1	Inhibition accelerated SMC differentiation	(114)
	ECs	TRPC3	Inhibits atherosclerosis	(115)
miR-27	Hepatic Cells	LPL	Regulates plasma and macrophage lipid levels and atherosclerosis	(116)
miR-29a	Plasma	Collagens I and III and Quaker	Regulates lipid uptake. Elevated plasma levels associated with increased carotid intima-media thickness in atherosclerosis	(117)
	VSMCs	Fbx7/CDC4	Regulates cell proliferation	(118)
miR-29b	VSMCs		Regulation of cell proliferation. Inhibition reduces in-stent restenosis	(119, 120)
			Elevated plasma levels associate with increased intima-media thickness	(117)
miR-33a/b	Liver, fibroblast, and macrophage cell lines	ABCA1	Regulates cholesterol homeostasis	(121–123)
miR-122	Liver		Decreases plasma cholesterol	(124)
	ECs	NPAS3	Regulates endothelial-to-mesenchymal transition and atherosclerosis	(125)
miR-126	ECs	Dlk1 V-CAM1	Enhances angiogenesis and endothelial repair	(126, 127)
miR-130	ECs	PPAR*γ*	Promotes inflammation and atherosclerosis	(128)
miR-132 and miR-133	Blood		Expression associates with subclinical atherosclerosis	(98)
miR-145	VSMCs	KLF4	Regulate VSMC differentiation. Downregulation contributes to atherosclerosis	(129)
miR-146a	VSMCs	KLF4	Promotes VSMC proliferation and neointima hyperplasia *in vitro*	(130)
	EPCs	Plk2	Increased expression associates with senescence and apoptosis	(131)
miR-148a	Hepatocytes	LDLR and ABCA1	Regulates circulating lipoprotein	(132)
	ECs	KLF5	Regulates inflammation and EC injury in atherosclerosis	(133)
miR-155	VSMC-derived exosomes	ZO-1	Regulates EC proliferation and permeability	(134)
miR-186-5p	Serum exosomes	LOX-1	Regulates lipid uptake by macrophages and atherosclerosis	(135)
miR-216a	ECs	SMAD3	Induces endothelial cell senescence and inflammation	(136)
miR-217	ECs	SIRT1	Increased expression associated with cellular senescence	(137)
	ECs	TLR4	Regulates endothelial-to-mesenchymal transition	(138)
miR-221 and miR-222	VSMCs	P27 and p57	Necessary for vascular smooth muscle cell proliferation and neointimal hyperplasia	(139)
	ECs	PGC-1α	Overexpression results in endothelial injury in atherosclerosis	(108)
miR-302a	Macrophages	ABCA1	Modulator of cholesterol homeostasis and atherosclerosis	(140)
miR-320b	Macrophages	ABCG1 and EEPD1	Regulator of cholesterol efflux	(141)
miR-365	Monocytes and serum	IL-6	Participates in coronary atherosclerosis	(142)
miR-483-5p	Serum		High serum levels associate with asymptomatic carotid artery stenosis	(143)
miR-451a	Serum		Expression significantly decreased in patients with atherosclerosis	(144)
miR-503-5p	Macrophages	SMAD1, 2 &7	Elevated in atherosclerosis patients	(145)

Perhaps unexpectedly, miRNAs have been found to be relatively stable and can readily be detected in body fluids including blood, urine and saliva. This is now believed to be predominantly due to their encapsulation within microvesicles and particularly exosomes (146). These are small (30–150 nm in size) membrane vesicles which originate from intracellular compartments and are secreted by most cells (147). They carry bioactive (tissue-specific) cargo from their host cell, including proteins, lipids and nucleic acids (notably miRNAs) and are involved in intercellular communication and thus a wide range of physiological processes (148). The biological components of exosomes can therefore influence both physiological and pathophysiological processes. Further, it has been shown that the molecular constituents of the exosomes (including the miRNAs they carry) are themselves modulated by disease states (and responses to treatments) (149). Accordingly, there is now considerable investigation into the clinical use of levels of specific miRNAs as biomarkers for disease onset and progression. In the case of atherosclerosis, an increasing number of circulating miRNAs have been identified whose levels have been shown to associate with a thickening of the arterial wall in the very early stages of the of the disease and it is hoped that these will prove diagnostically useful in future [reviewed in (150)].

### Therapeutic approaches to target dysregulated non-coding RNAs in atherosclerosis

The numerous causal associations between dysregulated miRNAs and the onset and progression of AVD (tabled above) has driven the use of miRNA-based therapeutic approaches to either supress or restore their misexpression (151). Thus, miRNA mimics and anti-miRNAs (antagomirs) have been used to counteract the functional pathological consequences of reduced or upregulated miRNA expression respectively. However, there are considerable challenges associated with their use. Unmodified, naked nucleic acid oligonucleotides are subject to degradation by serum nucleases and are immunoreactive. Considerable progress has been achieved in the development of chemical modifications to stabilise therapeutic oligonucleotides including the use of 2′-*O*-methyl (2′-OMe), phosphorothioate, 2′-*O*-methyoxyethyl (2′-MOE), 2′-fluoro (2′-F) and *N*,*N*–diethyl–4-(4–nitronaphthalen–1–ylazo)- phenylamine (ZEN) modifications and locked nucleic acid (LNA) technologies. These are all aimed at increasing the stability and reducing the immune recognition of oligonucleotides while maintaining their binding affinity and specificity and functional efficacy [reviewed in (151)].

Other major challenges in delivering small RNA therapeutics include the specificity with which these molecules can identify and associate with their target cell(s) and the efficiency with which they can cross cellular membranes (152). In this regard there is increasing interest in the use of exosomes as vehicles for their delivery, which have inherent cell-type specificity and are naturally taken up by the target cell by endocytosis (153). Typically, such vesicles are isolated and purified from cultured cells *in vitro* before injecting them either into the circulation or directly into the target site (153). As required, the cells generating the exosomes in culture can be engineered to overexpress the desired small RNAs, for example by transfection or transduction, to become encapsulated into the secreted exosomes.

Communication between different cell types, mediated by exosomes, has increasingly been demonstrated to be important in both the prevention and the progression of atherosclerosis, dependent upon the physiological state of the donor cell [reviewed in (154)]. Exosomes generated from a variety of differentiated cell types have been used to deliver (usually detrimental) miRNAs in experimental studies of atherosclerosis, evidencing proof of principle. These are typically cells that are associated with the formation of atherosclerotic plaques and include macrophages, ECs and VSMCs among others (154). The functions targeted by the miRNAs in these studies usually represent different components of the atherosclerotic process such as inflammation, lesion formation or cholesterol biosynthesis. For example, purified exosomes, secreted by human aortic smooth muscle cells, which had been engineered to overexpress miR-155, were shown to transfer their miR cargo to endothelial cells within the aorta and promote plaque formation in atherosclerotic-prone (ApoE-/-) mice after injection into the tail vein (135). Endothelial-derived exosome cargos have been shown to modulate both VSMC plasticity and monocyte/macrophage phenotype. Accordingly, miR-92a secreted from ECs into exosomes was shown to regulate the contractile-to-synthetic phenotypic switch of VSMCs, contributing to arterial stiffness (155) and to enhance the proinflammatory responses and low-density lipoprotein uptake of cocultured macrophages (156). The ability of exosomes derived from macrophages to affect the VSMC phenotype *in vivo* has also been demonstrated. Thus exosomes isolated from nicotine-treated macrophages induced VSMC proliferation and migration via the miR-21-3p-dependent targeting of PTEN *in vitro*, while *in vivo* they promoted the generation of atherosclerotic lesions in mice (157). Going forward, and based on these studies, the use of genetically engineered exosomes to deliver miRNAs known to be beneficial in the treatment of atherosclerosis is therefore very promising. It should be noted that cell-free methodologies can also be used to load vesicles with miRNAs after their isolation. Thus, miRNAs can be introduced into exosomes that have been isolated directly from biological fluids, by a number of chemical and physical methods including transfection and electroporation. miRNAs were shown to be introduced successfully into exosomes isolated from a number of different sources, and furthermore these exosomes retained their abilities to deliver the miRNAs to the target cell (158).

### Therapeutic approaches using stem cell-derived exosomes

Stem cell transplantation also represents a promising strategy to repair and regenerate diseased tissue in CVD, including atherosclerosis and has been shown to be effective in animal models (159). However, this approach has limitations due to the compromised survival and differentiation, together with potential immunoreactivity of (allogenic) stem cells (160). The use of stem cell-derived exosomes represents an acellular therapeutic strategy (which avoids the issues often seen with stem cell transplantation) and atherosclerosis is now being widely investigated using stem cell-derived exosomes carrying miRNAs (161). Further, much evidence now suggests that many of the beneficial effects of stem cell administration can, in fact, be attributed to the secretion of paracrine factors by the transplanted cells, including exosomal miRNAs (162). Thus, exosomes derived from progenitor cells, such as mesenchymal stem cells (MSCs), endothelial progenitor cells (EPCs), bone marrow derived macrophages (BMDM) and platelets have been shown to express endogenous miRNAs that ameliorate and inhibit atherosclerosis progression [reviewed in (154)]. For instance, MSC-derived exosomes containing miR-21a-5p were shown to attenuate atherosclerosis in the ApoE-/- mouse model, via promoting M2 macrophage polarization and infiltration (163). In another recent study, miR-100-5p in human umbilical cord mesenchymal stem cell-derived exosomes was shown to reduce plaque formation in atherosclerosis-prone mice via alleviation of eosinophil inflammatory responses (164). The treatment of diabetic-associated atherosclerotic mice with EPC-derived exosomes was also demonstrated to be beneficial in reducing the disease, although in this study the therapeutic activity was not assigned to a specific miRNA (165).

### Conclusions and future prospects

The development of vascular disorders including atherosclerosis has increasingly often been demonstrated to be driven by environmentally-acquired, aberrant changes in DNA methylation. In some cases these may be due to (clonal expansion of) somatic mutations within the epigenetic modifying enzymes, *Tet2* and *Dnmt3a*. Genetic editing, to reverse mutations known to be causal in the development of atherosclerosis represents a promising stem cell-based therapeutic strategy in the elusive struggle to reverse the progression of atherosclerosis. *Tet2* and *Dnmt3a* may therefore be potential therapeutic targets for gene editing in this context. Alternatively, epigenetic editing, involving the CRISPR/Cas9-dependent guiding of epigenetic modifiers to target the aberrant methylation marks themselves represents another potential therapeutic strategy.

The misexpression of specific miRNA(s), when shown to be causal in atherosclerosis development, also represents a promising therapeutic target to treat the disease. Genetically-programmed cell-based therapies involving the generation of exosomes loaded with specific small RNA cargo are therefore also being developed. Further, the clinical use of (naturally-occurring) exosomes expressing therapeutically-beneficial miRNAs, isolated from stem cell populations offers great promise.

Both the above approaches have significant inherent challenges to overcome and will rely on a fuller understanding of the methylation/miRNA changes involved, their causal relationship with the disease, and the involvement of specific cell-types in atherosclerosis pathology. This will be necessary both to inform the epigenetic changes to target and the choice of stem cell to engineer.
